# Localization of a Power-Modulated Jammer

**DOI:** 10.3390/s22020646

**Published:** 2022-01-14

**Authors:** Pietro Tedeschi, Gabriele Oligeri, Roberto Di Pietro

**Affiliations:** Division of Information and Computing Technology (ICT), College of Science and Engineering (CSE), Hamad Bin Khalifa University (HBKU), Doha P.O. Box 5825, Qatar; goligeri@hbku.edu.qa (G.O.); rdipietro@hbku.edu.qa (R.D.P.)

**Keywords:** jamming, jamming localization, power-modulated jammer, security, wireless

## Abstract

Jamming is a malicious radio activity that represents a dreadful threat when employed in critical scenarios. Several techniques have been proposed to detect, locate, and mitigate jamming. Similarly, counter-counter-jamming techniques have been devised. This paper belongs to the latter thread. In particular, we propose a new jammer model: a *power-modulated* jammer that defies standard localization techniques. We provide several contributions: we first define a new mathematical model for the *power-modulated* jammer and then propose a throughout analysis of the localization error associated with the proposed power-modulated jammer, and we compare it with a standard *power-constant* jammer. Our results show that a power-modulated jammer can make the localization process completely ineffective—even under conservative assumptions of the shadowing process associated with the radio channel. Indeed, we prove that a constant-power jammer can be localized with high precision, even when coupled with a strong shadowing effect (σ ≈ 6 dBm). On the contrary, our power-modulated jammer, even in the presence of a very weak shadowing effect (σ < 2 dBm), presents a much wider localization error with respect to the constant-power jammer. In addition to being interesting on its own, we believe that our contribution also paves the way for further research in this area.

## 1. Introduction

Jamming is a malicious radio activity carried out with the purpose of disrupting wireless communications. Jamming can be considered a physical-layer denial-of-service attack, where the adversary intentionally prevents one or more wireless communications by affecting the capability of the receiver(s) to correctly detect and receive a transmitted message [1]. Deploying jamming is extremely easy and cost-effective. Indeed, cost-effective software-defined radios (SDRs) make the design and implementation of a jammer straightforward, are able to eavesdrop on the radio spectrum, identify the target frequency to be jammed and finally, transmit a disrupting signal that prevents the receiver discriminating the purported one. Although jamming can be used by network nodes to exclusively guarantee wireless communications capabilities to a specific set of (ally) devices, the most practical and dangerous purpose of jamming is to completely disrupt the communications in a target area. Typical examples of critical targets are airports, sensitive infrastructures, or tactical military scenarios [2]. Jamming mainly involves three research areas: (i) *detection*; (ii) *localization*; and (iii) *mitigation* [3,4]. A jammer, by definition, is exposed—in order to disrupt the communications, it has to transmit; therefore, the jammer has to deploy smart techniques to avoid detection and localization. Common techniques for going undetected imply shaping the jamming signal in a way to make it indistinguishable from “normal” interference. Moreover, as per the vast majority of denial-of-service techniques, jamming cannot be completely prevented. Indeed, jamming mitigation involves the combination of different techniques to evade the jamming signal [5,6], thus enabling the receiver (and the transmitter) to work in a clear portion of the spectrum. Finally, jammer localization techniques resort to the estimation of the received signal strength (RSS) to infer the distance to the jammer (ranging) [7]. When multiple collaborating sources perform such a ranging technique, the position of the transmitter (jammer) can be precisely estimated.

**Contribution.** In this paper, we propose a new type of jammer, i.e., the *power-modulated* jammer. Our intuition is that a jammer that randomly changes its transmission power can make the localization process much harder (even impossible in some cases). In particular, after defining the power-modulated jammer model, we test a standard jammer (constant power) against a power-modulated jammer in the presence of a network of nodes running a classical localization technique, i.e., linear least square (LLS). Our findings confirm our intuition. In particular, we show that the power-modulated jammer—when compared against the constant-power jammer—makes the localization process particularly difficult, even in the presence of a high density of sensing nodes (10 nodes in a 40×40 m area) and under conservative assumptions of the (shadow) fading process of the wireless channel (σ<3 dBm).

**Paper organization.** The remainder of this paper is organized as follows. In Section 2, we review background information and related work, while in Section 3, we introduce the system and the adversary model discussed in this work. In Section 4, we compare the new proposed jammer model (power modulated) with the classical one (constant-power) in terms of localization error. We provide a comprehensive discussion on the performance of the proposed jammer model and some potential mitigation technique in Section 5, while in Section 6, we draw conclusions and discuss future work.

## 2. Related Work

Several contributions have been proposed to localize a jammer by taking into account different attack models. The algorithms can be mainly classified into two categories: (i) *range-based,* that estimate the distance from the jammer leveraging physical layer properties of the transmitted signal; and (ii) *range-free*, that leverage information about the network topology and the geometric features of the jammed area. The typical metrics employed to localize a jammer are: Jamming Signal Strength (JSS), Packet Delivery Ratio (PDR), Neighbor List Change (NLC) and the network topology information [8]. Pelechrinis et al. [9] proposed a distributed lightweight and generic jammer localization technique that leverages the gradient descent minimization algorithm. The authors analyzed the spatial effects of the jammer; in particular, they observed that the lower the PDR is, the closer we are to the jammer. Wang et al. [10] described a solution to estimate the jammer position by requesting the nodes in the jammed area to increase the power transmission until they are able to transmit and receive messages. The node with the higher value of the received JSS in the network will allow the better estimation of the jammer position. In [11], the authors leveraged the Received Signal Strength (RSS) metric in order to localize a jammer in a jammed area. The proposed protocol requires that the nodes are able to increase the power transmission in the jammed area until they can exchange measurement data about the estimation of the jammer position. A few other works proposed a solution that takes into account neighbor changes. For instance, in [12], the authors introduced a jammer localization algorithm that leverages the neighbor changes in the communication range and adopts the least-squares method, while others in [13] presented a virtual-force-based jammer localization algorithm scheme that exploits the average jammed nodes position data, i.e., the coordinates. In [14], Liu et al. presented a jamming localization technique that allows localizing multiple jammer devices by leveraging the changes in the network topology. The proposed framework partitions the network topology into clusters and estimates the positions of multiple jammers. On the other hand, Sun et al. [15] highlighted a technique that exploits the collaboration of sensor nodes that share their locations at the border of the jammed area to compute the coordinates of the jammer. Each node computes the convex hull for the set of victim nodes based on their coordinates and finally extracts the smallest circle that covers all nodes in the convex hull to identify the jammer position. Tedeschi et al. [16] described an autonomous jamming-assisted navigation system that allows a drone to accomplish its mission by locating the jammer and leveraging the discovered position to support navigation. Cheng et al. [17] designed an algorithm, namely double-circle localization (DCL), based on minimum bounding circle (MBC), maximum inscribed circle (MIC) and the network topology, without considering any other features of the jammed area. The authors combined the centers of the two circles as the estimated jammer position. Finally, in order to evaluate the accuracy, the authors leveraged the Euclidean distance between the estimated and true position of the jammer as the localization error. The authors in [18] presented two algorithms for jammer localization: (i) a multi-cluster localization algorithm; and (ii) X-rayed jammed-area localization. The M-cluster algorithm groups jammed nodes with a clustering algorithm, and each jammed-node group is used to estimate one jammer location. The X-ray algorithm relies on the skeletonization of a jammed area and uses the bifurcation points on the skeleton to localize the jammers. Bhamidipati and Gao [19] highlighted a solution to localize multiple jammers, with the analysis of the variation in the front-end signal power, recorded by the Unmanned Aerial Vehicles (UAVs) on-board GPS receivers in the network. Basically, the authors leveraged a Gaussian mixture probability hypothesis density filter over a graph framework and the Levenberg–Marquardt algorithm as a minimizer.

Despite the large number of research contributions on jammer localization, there are no studies based on a scenario that adopts a power-modulated jammer—such as the one introduced in this paper.

## 3. Scenario and Assumptions

**Figure 1:**
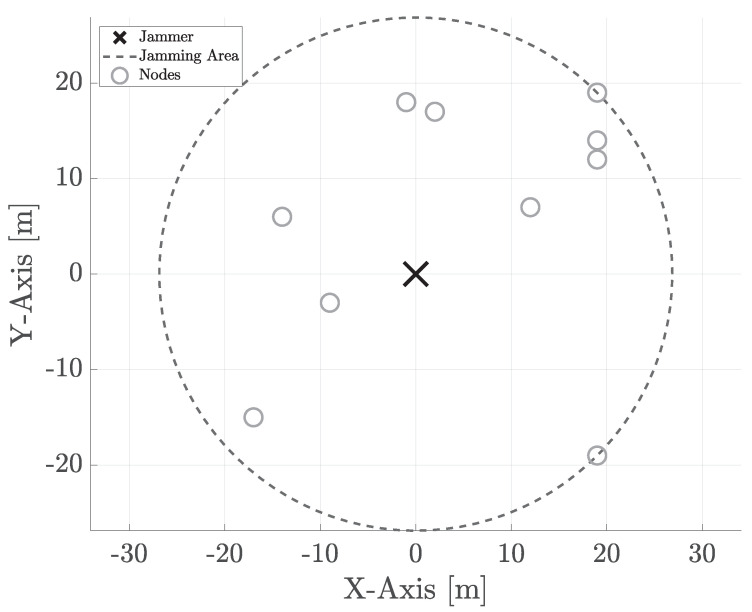
A typical random deployment: 10 nodes and the jammer (black cross) at the center of the scene. The black dashed circle represents the jamming area.

Let us consider a playground constituted of a set of randomly deployed sensing nodes $\left\{n_{1},\ldots ,n_{N}\right\}$, where *N* spans between 3 (minimum number of sensors to perform localization) and 10. Moreover, we assume the presence of a jammer at coordinates [0, 0] (center of the scene). Figure 1 shows typical deployment in an area of 40×40 m. The jamming power is reset at each simulation-run to fit the node deployment, thus covering all the communication links. In particular, we assume that the jammer is deployed at the center of the scene without being aware of the actual position of the nodes. However, before starting the jamming activity, it eavesdrops the radio spectrum to infer the relative position of the furthest node, thus subsequently adapting its jamming power.

**Figure 2:**
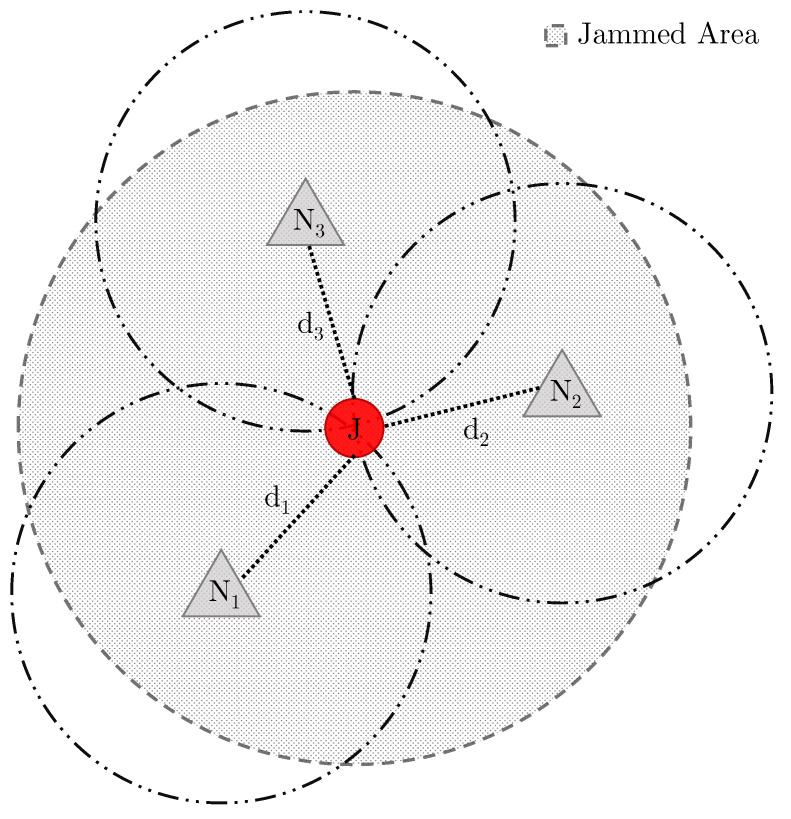
Jammer localization by three-lateration: 3 sensors collaborating to estimate the jammer position.

We assume that the jammer is able to continuously transmit a (either constant or power-modulated) jamming signal. Moreover, we assume that the sensors are able to sense the jamming signal and resort to a non-jammed communication channel to communicate with each other. Indeed, sensors collaborate to locate the jammer: without loss of generality, we can assume that one of the sensors collects readings from the other ones and processes them to generate the approximated jammer location. Assuming the existence of an out-of-band communication channel to exchange information useful to locate the jammer—while we aware is not a realistic scenario—it is nevertheless a worst case for the jammer, and this is specifically what is needed to compare the performance of the jammer in terms of their capacity of delaying/denying localization. We adopted a classical multi-lateration approach to estimate the position of the jammer [20], and we also conducted an extensive simulation campaign using MATLAB©2021b. We remark that the objective of this paper was not to perform jamming localization but to discuss the impact of power modulation on jamming localization, especially in comparison with a standard power-constant jammer. Figure 2 shows typical three-lateration (3 sensors collaborating for jamming localization) to estimate the jammer position. Sensors $\left\{n_{1},n_{2},n_{3}\right\}$ estimate the distance to the jammer by mapping the received jamming signal strength to a distance value. By combining the three distances $\left\{d_{1},d_{2},d_{3}\right\}$, it is possible to approximate the position of the jammer.

**Path loss model.** We adopted the standard log-normal path loss model as depicted by Equation (1) where $R_{x}\left(t\right)$ is the received signal strength, $T_{x}\left(t\right)$ is the jamming power, γ is the path loss exponent, and $d_{0}$ is a reference distance at which the path loss $PL(d_{0})$ is measured. Finally, $X_{\sigma }$ is a log-normal random variable with a mean μ=0 and standard deviation σ taking into account the shadowing effect of a flat-fading channel:

(1) $$R_{x}\left(t\right)=T_{x}\left(t\right)-PL\left(d_{0}\right)-10\gamma \log\frac{d}{d_{0}}-X_{\sigma }\left(t\right)$$

In this paper, we considered a jammer that is randomly varying the jamming power $T_{x}\left(t\right)$ as a function of time *t*. Moreover, we assume that the shadowing effect being modeled by the random variable $X_{\sigma }\left(t\right)$ is independent of the jamming power variations, i.e., $T_{x}\left(t\right)$ and $X_{\sigma }\left(t\right)$ are independent random variables.

**Jamming power.** As previously mentioned, the jamming power is the result of a trade-off between the actual available transmission power of the jammer and the power requested to jam the whole area, i.e., the jammer wants to jam all the playground with a minimum amount of transmission power. Without loss of generality, we assume a node receiver sensitivity $S_{rx}$ is equal to $S_{rx}=-80$ dBm, and we dynamically set the minimum transmission power as a function of the deployment as depicted by Equation (2):

(2) $$\begin{array}{c} T_{x}{|}_{min}=S_{rx}+PL\left(\max_{i\in \{1,\ldots ,N\}}d\left(J,n_{i}\right)\right)+\\ PL\left(\min_{\left(i,j)\in \{1,\ldots ,N\right\}}d\left(n_{i},n_{j}\right)\right) \end{array}$$

The first packet loss term (*PL*) takes into account the maximum distance between the jammer and the nodes in the playground, while the second term considers the power margin (guard) to enable the communication between the closest possible pair of nodes. By considering Equation (2), we are guaranteed that the jammer transmission power overwhelms all the ongoing communications in the playground.

**Ranging.** Assuming the propagation model introduced by Equation (1), the approximated distance $\tilde{d}$ between the sensor and the jammer can be computed as described by Equation (3):

(3) $$\tilde{d}=10^{\frac{T_{x}\left(t\right)-R_{x}\left(t\right)}{10^{\gamma }}}.$$

To ease exposition, we chose $d_{0}=1$ m and $PL(d_{0})=0$ dBm. Moreover, we assume that $X_{\sigma }\left(t\right)=0$ since we consider the case of averaging multiple consecutive channel readings, thus being able to mitigate the shadowing effect.

**Localization.** The estimated distances $\left\{d_{1},\ldots ,d_{N}\right\}$ from the ranging process are combined to generate an approximated position for the jammer $\left[x_{J},y_{J}\right]$. We first compute a linearization of the problem by choosing one sensor ($x_{N},y_{N}$) and its related distance to the jammer ($d_{n}$) as a reference, and by subtracting it to the n−1 equations obtaining a system of n−1 equations in the form Az=b, yielding:

$$A=-2\times \left(\begin{array}{cc} \left(x_{1}-x_{n}\right) & \left(y_{1}-y_{n}\right)\\ \left(x_{2}-x_{n}\right) & \left(y_{1}-y_{n}\right)\\ \vdots & \vdots \\ \left(x_{n-1}-x_{n}\right) & (y_{n-1}-y_{n}), \end{array}\right)$$

$$b=\left(\begin{array}{c} x_{n}^{2}+y_{n}^{2}-y_{1}^{2}-x_{1}^{2}+d_{1}^{2}-d_{n}^{2}\\ x_{n}^{2}+y_{n}^{2}-y_{2}^{2}-x_{2}^{2}+d_{2}^{2}-d_{n}^{2}\\ \vdots \\ x_{n}^{2}+y_{n}^{2}-y_{n-1}^{2}-x_{n-1}^{2}+d_{n-1}^{2}-d_{n}^{2} \end{array}\right)$$

We can now estimate the location of the jammer by solving the system Az=b with the Linear Least Square (LLS) method, as shown in Equation (4):

(4) $$z={\left[x_{J},y_{J}\right]}^{T}={\left(A^{T}A\right)}^{-1}A^{T}b$$

### Constant and Power-Modulated Jammer

**Figure 3:**
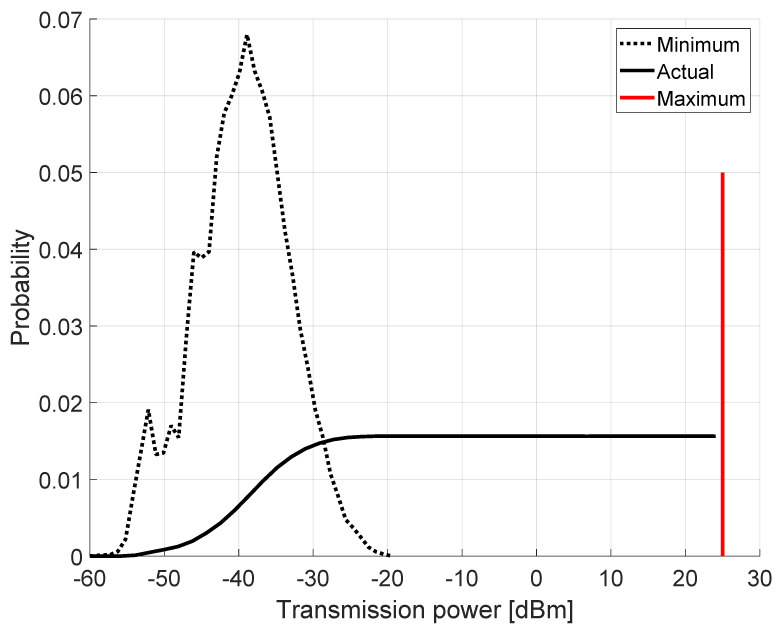
Power-modulated jammer: the minimum transmission power is opportunistically chosen in order cover the whole area.

Classical jamming localization problems assume a jammer that keeps transmitting over time with the same power—this one being chosen to disrupt all communications in the area. We refer to such a behavior as *constant-power jammer*. Although the jammer can implement different strategies to stay as stealthy as possible, it cannot reduce the transmitting power under a certain threshold, i.e., the power to cover the whole playground. Therefore, the only significant source of uncertainty to jammer localization comes from the shadowing effect, i.e., the random variable $X_{\sigma }$ from Equation (1). As will be subsequently clarified (as well as in related works), this can be mitigated by increasing both the number of sensing nodes and the readings of the received signal strength at each node. Our intuition to make jammer localization more difficult is to adopt a *power-modulated jammer* that randomly changes its transmission power $T_{x}\left(t\right)$ as a function of time *t*. The jamming power $T_{x}\left(t\right)$ is uniformly chosen in the interval $T_{x}\left(t\right)\in \left[T_{x}^{min},T_{x}^{max}\right]$. As previously discussed for the constant-power jammer, $T_{x}^{min}$ is chosen in order to cover the whole playground, while $T_{x}^{max}=25$ dBm. The maximum transmission power can be chosen as a function of different factors. As will become clear in the following, increasing $T_{x}^{max}$ makes the jammer’s position more difficult to estimate; nevertheless, this significantly affects the jammer’s energy budget. Figure 3 shows the transmission power strategy implemented by the jammers. The constant-power jammer sets the transmission power to the strict minimum (dotted line in Figure 3) necessary that allows the full coverage of the playground. Indeed, given the random deployment experienced in every simulation run, the minimum transmission power varies between −60 and −20 dBm. Conversely, the power-modulated jammer chooses the minimum transmission power in the same way as the constant jammer, but it also set a maximum transmission power as depicted by the solid red line in Figure 3 being equal to 25 dBm. Finally, the actual transmission power is randomly chosen from the current minimum and the maximum as depicted by the solid black line in Figure 3.

## 4. Jammer Localization

**Figure 4:**
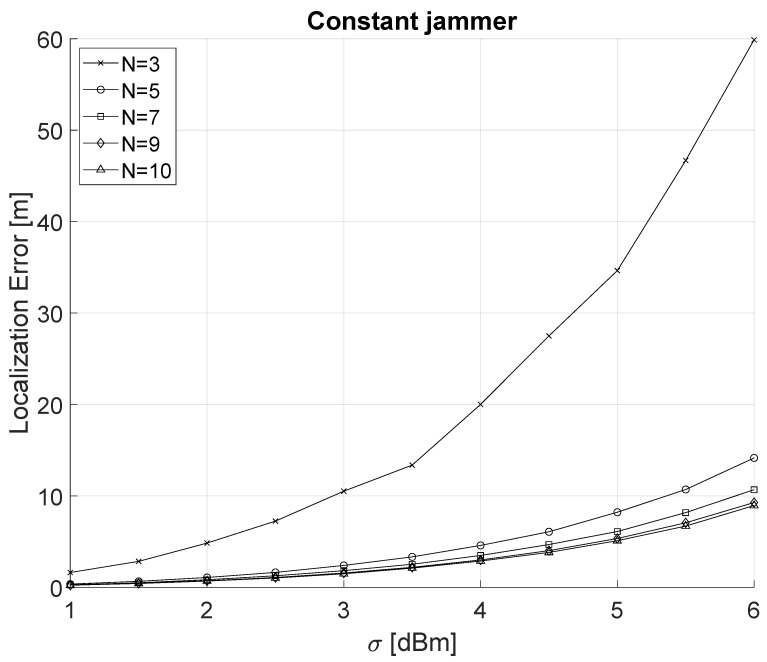
Localization of a constant jammer as a function of σ (1 dBm ≤ σ ≤ 6 dBm) and the number *N* of sensing nodes (3≤N≤10).

In this section, we compare the previously introduced localization algorithm (LLS) against both a constant and a power-modulated jammer. We start our analysis from the constant-modulated jammer considering the shadowing effect with a variance σ in the range (1 dBm ≤σ≤ 6 dBm) and a number *N* of deployed sensors (3≤N≤10). Figure 4 shows the localization error as a function of σ and *N* for the case of a constant jammer. The localization error was computed as the Euclidean distance between the actual [0, 0] and estimated jammer position. Finally, our results take into account the average value of 5000 simulation runs and an average of 100 jamming signal readings.

We highlight that the maximum localization error is approximately 60 m for the case N=3 and σ=6 dBm. Indeed, this is the worst-case scenario for a localization setting, i.e., the minimum number of nodes and maximum variance due to shadowing. When the number of sensing nodes increases (N>3), and assuming smaller values for σ, i.e., σ<4 dBm, the localization error is reduced to less than 4.6 m.

**Figure 5:**
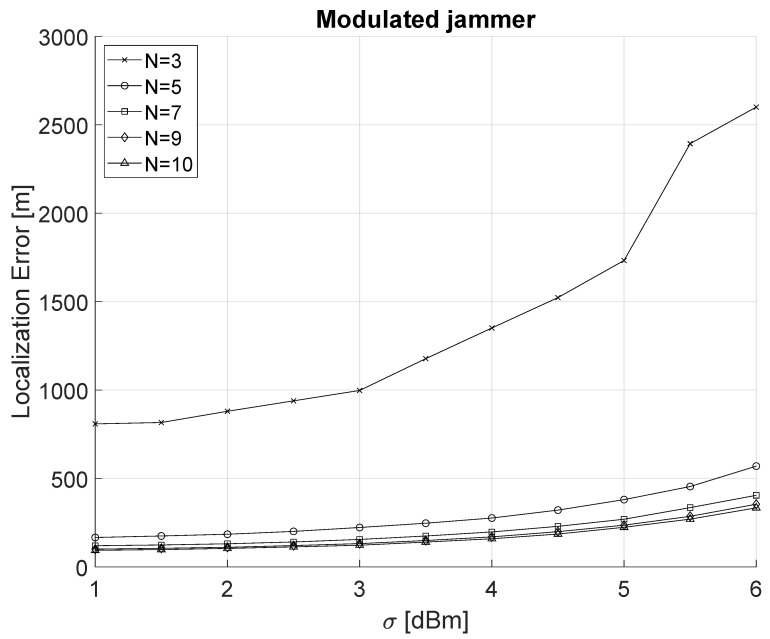
Localization of a power-modulated jammer as a function of σ (1 dBm ≤ σ ≤ 6 dBm) and the number *N* of sensing nodes (3≤N≤10).

Figure 5 shows the localization error of a power-modulated jammer varying both σ and the number of sensing nodes in the network. The localization error for the modulated-jammer turns out to be significantly higher than the constant jammer. Even assuming the best configuration parameters, i.e., N=10 and σ=1 dBm, the localization error is approximately 93 m. Moreover, we observe a significant improvement between N=3 and N=5 deployed nodes, while further increasing the number of nodes (N>5) does not give any significant advantage in the localization process.

**Figure 6:**
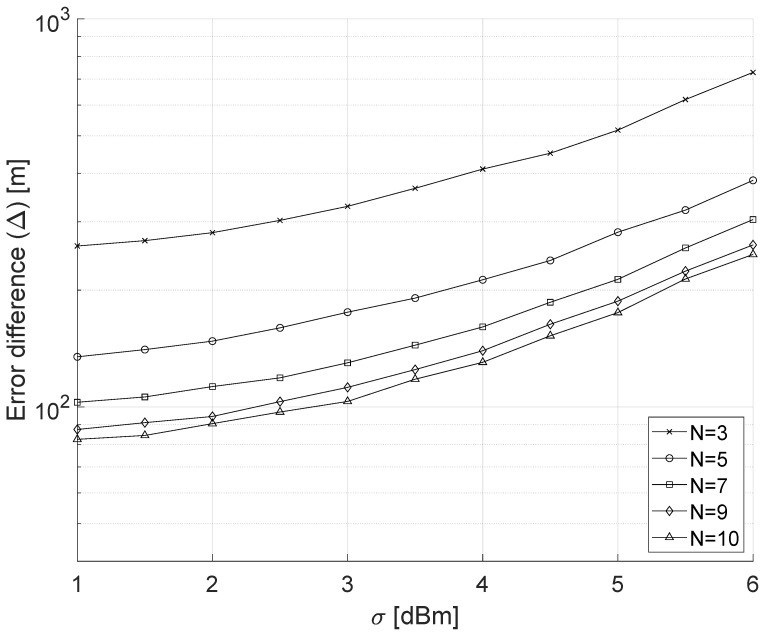
Comparison between the modulated-power and constant-power jammer: localization error as a function of σ while the number of nodes in the playground spans between N=3 and N=9.

We now consider the difference Δ between the localization error of the modulated and the constant-power jammer. Figure 6 shows Δ as a function of 1 dBm ≤σ≤ 6 dBm and N∈{3, 5, 7, 9, 10}. As previously observed, the number of nodes mitigates the uncertainty associated with the jammer position, i.e., when σ=3 dBm, Δ varies between approximately 328 m (N=3) and approximately 112 m (N=9). Moreover, we observe how the combined effect of σ and the modulated power can significantly hinder the localization; indeed, the case N=3 and σ=1 dBm has approximately the same performance as N=9 and σ=6 dBm. Finally, we observe how even under conservative assumptions for σ, i.e., σ<3 dBm, increasing the number of nodes does not significantly affect the localization performance, i.e., the error difference does not significantly increase when moving from N=9 to N=10. Lastly, we consider the number of readings of the jamming signal. As previously discussed, we considered 100 readings for all the performance. In the following, we consider 5000 readings, and we estimate the relative error $\epsilon_{r}$ for the modulated jammer as follows:

(5) $$\epsilon_{r}=\frac{E_{5000}-E_{100}}{E_{100}}$$

where $E_{5000}$ is the localization error for the modulated jammer assuming 5000 readings, while $E_{100}$ is the localization error for the modulated jammer assuming 100 readings (baseline case). We observe that increasing the number of readings affects the localization error depending on σ; indeed, the localization relative error goes from approximately 0 to −25% when σ spans between 1 dBm and 6 dBm. Higher values of readings mitigate the randomness of the shadowing process, thus becoming more effective when σ is bigger. Finally, we would like to stress that increasing the number of nodes only very marginally affects the performance of the localization process—namely the four curves crossing over at several data points, and partially overlapping for a few segments of interest.

## 5. Discussion

**Figure 7:**
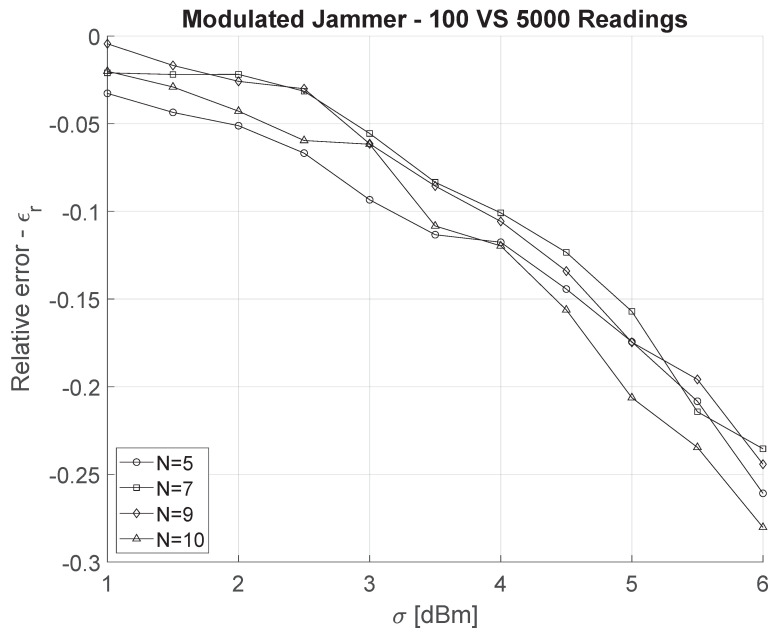
Comparison between 100 and 5000 readings of the jamming signal as a function of σ and the number of nodes.

The localization error associated with the position of a jammer (either constant or power-modulated) mainly depends on three factors: the randomness of the channel (shadowing), the capability to observe the jamming signal (number of readings), and finally, as proven by this contribution, the behavior of the jammer (power modulation). Power modulation requires more energy with respect to a constant jammer that can calibrate its power to the minimum requested to cover the target area. Nevertheless, we highlighted how a power-modulated jammer can trade-off the increased transmission power with a higher localization error—in many cases, the error is 10 times the width of the playground, making the approximated position of the jammer completely inconsistent with the actual one. By considering the constant-jammer as the baseline scenario, the power-modulated jammer introduces significant uncertainty in the localization process (recall Figure 6). Even under conservative assumptions of shadowing (σ≤3 dBm), the position of the modulated-power jammer cannot be identified, i.e., Δ>100 m, in a playground of 40×40 m. We observe how increasing the number of nodes in the playground is effective up to N=10, while subsequently, Δ does not significantly decrease. Finally, the number of readings can help the localization process under strong shadowing (σ>3 dBm); nevertheless, from combining Figure 5 with Figure 7, it emerges that the gain in localization accuracy is still not sufficient to pinpoint the jammer: adopting 5000 readings makes the localization process 25% more accurate than in the case of 100 readings; however, the localization error remains excessively high, i.e., at approximately 400 m, when σ≈6 dBm.

**Mitigation and future work.** Locating a power-modulated jammer turns out to be a challenging problem. A naive solution might involve an ultra-dense sensing network, but this solution might be impractical in many scenarios and not scaling up with the area to be protected by the action of the jammer. All our considerations mainly focus on sensing devices featuring an isotropic antenna. While this set-up is very effective in the localization of a standard transmitter (such as a constant jammer), it turns out to be much less ineffective against a *power-modulated* jammer. Conversely, a directive antenna might be much more effective for determining the direction of the jamming signal, thus making multiple collaborating directive antennas able to effectively locate the power-modulated jammer. We observe how, in this case, the localization algorithm should not be based on multilateration but on triangulation. Another consideration might be concerned with detecting the actual presence of a power-modulated jammer in order to subsequently deploy an ad hoc strategy for its localization. Indeed, we can assume that the sensing nodes monitor the spectrum, and they can detect and identify the jamming signal. The sensing nodes might be able to model the random process behind the power-modulation, thus pre-conditioning the input to the localization algorithm. Finally, we would like to highlight that different localization algorithms might be affected in different ways by the modulated jammer—and in turn, the associated localization error. Our contribution aims at defining the theoretical framework for a new jammer strategy willing to evade the localization process. The choice of proper localization algorithm is only one of the parameters that can mitigate the evasion and we leave this wider discussion to future works.

## 6. Conclusions

In this paper, we introduced a new jammer model: the *power-modulated* jammer. We proved that a jammer that randomly modulates its transmission power can make the localization process quite ineffective. We compared our new jammer model with a classical one (*constant-power* jammer), and we highlighted how a modulated jammer is much more difficult to locate than a constant one even under very unbalanced conditions of the shadowing effect, i.e., a weak shadow for the modulated jammer versus a strong shadow for the constant one. Furthermore, the localization accuracy of the proposed jammer model does not improve sensitivity while increasing the sensing nodes or the sampling frequency—a testament to the unique features of the introduced model. Finally, we also discussed future work, as well as preliminary mitigation strategies. We also released the source code of our proof of concept [21], enabling the interested community to verify our findings, as well as to foster further research in the domain. We believe that the novelty of the proposed model and the excellent performance achieved herein could attract the attention of the scientific and industrial community for future research in this area.

## Data Availability

Not applicable.
